# Postprandial Glucose Levels Are Better Associated with the Risk Factors for Diabetes Compared to Fasting Glucose and Glycosylated Hemoglobin (HbA1c) Levels in Elderly Prediabetics: Beneficial Effects of Polyherbal Supplements—A Randomized, Double-Blind, Placebo Controlled Trial

**DOI:** 10.1155/2019/7923732

**Published:** 2019-04-15

**Authors:** Jingfen Zhu, Guoqiang Xing, Tian Shen, Gang Xu, Yun Peng, Jianyu Rao, Rong Shi

**Affiliations:** ^1^Department of Community Health and Behavior Medicine, School of Public Health, Shanghai Jiao Tong University, Shanghai 200025, China; ^2^The Affiliated Hospital and the Second Clinical Medical College of North Sichuan Medical College, Nanchong Central Hospital, Nanchong 637000, China; ^3^Lotus Biotech.com LLC, John Hopkins University-MCC, 9601 Medical Center Drive, Rockville, MD 20850, USA; ^4^Sanlin Community Health Service Center, Pudong New District, Shanghai 200120, China; ^5^Department of Pathology and Laboratory Medicine, David Geffen School of Medicine, University of California, Los Angeles, CA 90095, USA; ^6^School of Public Health, Shanghai University of Traditional Chinese Medicine, Shanghai 201203, China

## Abstract

**Backgrounds:**

Prediabetes is a condition in which a person's blood glucose levels are higher than normal physiological levels but lower compared to patients with diabetes. Up to 70% of individuals with prediabetes will eventually develop diabetes. To date, there have been no pharmaceutical drugs to treat diabetes. It is believed that early diagnosis and nonpharmacological intervention for prediabetes are critical for effective prevention of diabetes. Most individuals with prediabetes remain undiagnosed even after being evaluated using the standard tests for fasting glucose (FG) and HbA1c. We investigated if postprandial glucose levels (2h-PG) were associated with pre/diabetes and if polyherbal supplements could be beneficial for individuals with prediabetes.

**Materials and Methods:**

100 elderly individuals with impaired 2h-PG or fasting glucose levels were recruited to receive either a 12-week supplement of GlucoVita (an antioxidative polyherbal formulation) (n=50) or placebo (n=50).

**Results:**

No baseline differences were observed for FG, HbA1c, or 2h-PG. Individuals who received a twelve-week administration of GlucoVita supplements had significantly reduced 2h-PG (8.15±1.67 versus 7.35±2.06 mmol/l, P<0.05) levels compared to individuals in the placebo group. In addition, HbA1c levels were lower in individuals who received GlucoVita (5.81±0.49 %) compared to the individuals in the placebo group (6.00±0.51%) (P=0.08) after 12-weeks. Stratified analysis, based on impaired fasting glucose (IFG), 2h-PG, metabolic symptom, and age, demonstrated that, after the 12-week intervention, HbA1c levels were significantly lower in the GlucoVita administered group compared to the placebo group (IFG subgroup; 5.85±0.46%, n= 27 versus 6.14±0.50, n=33, P<0.05) and the metabolic symptom-free subgroup (5.73±0.45%, n=23 versus 6.04±0.52%, n=24, P<0.05). GlucoVita also reduced FG in individuals with normal 2h-PG (6.37±0.27 versus 6.08±0.38 mmol/l, P<0.05). Baseline 2h-PG levels, but not HbA1c or FG levels, were significantly correlated with body weight, waist circumference, and BMI (r=0.25, P<0.05; r=0.31, P<0.01; r=0.22, P<0.05, respectively).

**Conclusion:**

2h-PG levels were better associated with body weight, waist circumference, and BMI risk factors compared to FG and HbA1c levels in elderly individuals with prediabetes. Polyherbal formulation GlucoVita supplements improved 2h-PG and HbA1c levels only in elderly individuals who were overweight but were symptom-free and under 65 years of age. Due to the small cohort size of this pilot study, future studies are required to validate our findings.

## 1. Introduction

More than 330 million individuals worldwide are estimated to have prediabetes and up to 70% will develop diabetes within a decade of initial diagnosis [1]. According to the US Centers for Disease Control (CDC), 84 million Americans (more than 1 out of 3) have prediabetes. Of these, 90% are unaware of their prediabetic status and up to 70% of them will eventually progress to diabetes [2]. The average risk of developing diabetes is approximately 5–10% per year in individuals with IFG or IGT, which is 10-15 times higher compared to individuals with normoglycemia.

Prediabetes is a condition in which a person's blood glucose level is higher than normal physiological levels but lower than individuals with diabetes. Prediabetes increases the risk of diabetes and cerebrocardiovascular disorders, which are the major causes of death worldwide [3].

Despite the worldwide prevalence of prediabetes and diabetes, many patients remain undiagnosed. This may be due to the current reference standards for fasting glucose (FG), or the inconvenience for individuals taking the 2h-oral glucose tolerance test (OGTT). In 2010, the American Diabetes Association approved the more expensive assay of glycated hemoglobin A1c (HbA1c) as one of the three tests for diabetes and prediabetes screening. However, recent studies have shown substantial discordance between results from FG, OGTT, and HbA1c, with OGTT probably being the more accurate diagnostic test for pre/diabetes associated with age, BMI, physical activity, income, education, race/ethnicity, etc. [4–6]. Conversely, recent studies have shown that biomarkers for food intake and nutrient status were associated with glucose tolerance status and development of diabetes in the elderly [7]. The utility of these new dietary biomarkers for determining pre/diabetes status has not been comprehensively evaluated in different ethnic populations.

Oxidative stress, inflammation, and peripheral insulin resistance lead to pancreatic beta cell overproduction; however eventual depletion of insulin secretion is thought to underlie the development of prediabetes and diabetes [8–13]. Overweight, being 45 years or older, and lower physically active are associated with type 2 diabetes and are also the main risk factors for prediabetes [2, 14]. Modification in lifestyle, increase in physical activity, having a healthy diet, and maintaining a healthy body weight can lower the risk of developing prediabetes and diabetes [15–17]. However, many people have difficulties to adhere to these recommendations.

Herbal supplements have long been used for disease prevention and treatment. Recent cellular and animal model studies have suggested that herbal supplements could improve glucose metabolism and protect from oxidative stress-induced diabetes [18–24]. However, several of these findings were deduced from animal studies and have not been replicated and validated in human studies. So far, only a few clinical studies have been performed to validate the claims of the effectiveness of herbal ingredients on prediabetes. Numerous studies have reported the antidiabetic and antioxidative activities of momordica charantia (bitter melon) extracts in diabetic animal models [25–32]; however, other studies have reported insufficient evidence for the effects of momordica charantia for type 2 diabetes mellitus [33–35].

A recent study showed that daily intake of 1g of cinnamon for 12 weeks reduced fasting blood glucose and HbA1c levels in middle-aged type 2 diabetic patients [36]. The antidiabetic effects of cinnamon were reported to increase glucose uptake, improve insulin sensitivity in peripheral tissues, improve glycogen synthesis in the liver, restore pancreatic islets dysfunction, slow gastric emptying rates, and improve diabetic renal and brain disorders through multiple signaling pathways, including the PPARs, PI3K/IRS-1, GLUT4, and Nrf2 pathways [37].

In this study we compared the predictive value of FG, 2h-PG, and HbA1c levels in elderly Chinese individuals with prediabetes and evaluated the effects of 12-week administration of GlucoVita supplements, a polyherbal formulation, on these three blood parameters using a double-blinded, randomized placebo control trial design.

## 2. Materials and Methods

### 2.1. Study Participants

Patient recruitment was initiated on September 2, 2011, and completed on October 12, 2012, with the last follow-up observation completed on January 7, 2012. Individuals were recruited from the Sanlin Community Health Service Center, Pudong New District, Shanghai, China. All study procedures were conducted in accordance with the Helsinki Declaration of 1975 and were approved by the Shanghai Jiao Tong University School of Public Health Institutional Review Board. Written informed consent was obtained from all study participants prior to enrollment into the study. This study is part of a series investigations into the effects of dietary supplements in old adults with chronic health conditions. Similar research methodologies have been used and reported in previous studies [38].

Subjects who met the first and one of the following two criteria were eligible for study participation.

*Inclusion Criteria*

1. Healthy males or females at least 50 years of age.
2. Fasting glucose (FG) levels between 6.1 and 7.0 mmol/d.
3. 2-hour postprandial glucose (2h-PG) levels between 7.8 and 11.1 mmol/d.

*Exclusion Criteria*

1. History of diabetes.
2. Administered glucose lowing medication over the last 30 days.
3. Other serious health conditions.

### 2.2. Randomization and Blinding

Enrolled study participants were randomly assigned to the GlucoVita treatment group or the placebo control group. The randomization was performed using a predetermined randomization code which was generated using a random number generator.

Study participants and physicians were double-blinded for the treatment. Of the 100 enrolled participants, 88 of them completed the 12-week follow-up period, which included 43 in the GlucoVita group and 45 in the control group. Five individuals in the GlucoVita group and 4 in the placebo control group withdrew from the study due to objections from their family members. Two individuals in the GlucoVita group withdrew from the study due to diarrhea and nausea and one individual in the placebo group withdrew from the study due to respiratory symptoms (coughing).

Study participants in the control group received similar-looking capsules in color-coded bottles (white bottles for GlucoVita and yellow bottles for placebo control). Neither the study participants nor the physicians, including the study principal investigator, had knowledge of the specific color codes until the end of the study. Both the GlucoVita tablets and the placebo control tablets were manufactured and supplied by GardaVita® Inc. (Costa Mesa, California, USA). Each study participant was instructed to take 1 tablet with meals, two times per day for 12 weeks, with a new batch of supplements dispensed every month during the follow-up sessions.

The key active ingredients of GlucoVita formulation includes extracts from* Momordica charantia*, (Bitter melon), Gymnema, Fenugreek (*Trigonella foenum-graecum*), Indian tinospora (*Tinospora cordifolia*), Kino tree, Bael tree, Neem (*Azadirachta indica*), Cinnamon (*Cinnamomum tamala*), and Cluster fig (*Ficus carica*). All of these extracts have been safely used in traditional medicine for centuries.

### 2.3. Demographic Information, Medical Examination, and Patient Follow-Up

The date of birth, gender, status of impaired fasting glucose (IFG), impaired postprandial glucose tolerance (I2hPG), body weight index (BMI), status of hypertension, dyslipidemia, and the presence of diabetes-related symptoms (polydipsia, polyphagia, hyperuresis, underweight, overweight, and others, 1=less or none, 2= moderate, and 3= most severe) were recorded for each study participant at the time of the initial screening before randomization. Physical examinations that focused on 4 levels of general health conditions (better than average; average; declining health status; frequently sick, 1=true, 2=most true, 3=unsure, 4= most false, and 5= false) were evaluated for all study participants by a physician. A standard form was used to screen study participants and they were asked to return to the clinic on a weekly basis for the first month and on monthly basis for the remaining months. During follow-up, the physicians answered any concerns and evaluated compliance/adherence and recorded adverse events. Study participants were then resupplied with a new bottle of tablets.

### 2.4. Statistics Analysis

EpiData 3.1 software was used for data entry and SPSS 20 software was used for statistical analysis. Group data were presented as mean ± standard deviation or median ± Quartile Range (QR). Differences between the GlucoVita and placebo groups were compared using Student's t-test for quantitative variables with normal distribution or Chi-square for categorized variables. Appropriate stratification analysis was performed to control for confounding factors. Correlations between the variables were also determined for each of the subgroups. P<0.05 denoted statistical significance. All p-values reported were 2-sided.

## 3. Results

### 3.1. Demographic Characteristics

The baseline information on age, gender, glucose status, body weight, hyperlipidemia, hypertension, and diabetes-related symptoms is shown in Table 1. There were 17 males (39.5%) and 26 females (60.5%) in the GlucoVita group and 15 males (33.3%) and 30 females (66.7%) in the placebo group. The gender distribution between the two groups was not significantly different (*χ*^2^=0.36, P>0.05). The average age of all participants was 63.29±7.84, and no significant age differences were present between the GlucoVita group (63.57±8.11, yr) and the placebo group (63.03±7.66, yr) (t=0.32, P>0.05).

There were no distribution pattern differences between the GlucoVita and placebo groups for impaired fasting glucose (IFG) (37.2% versus 35.6%, P>0.05) and impaired 2h-postprandial glucose (I2hPG) (37.2 % versus 26.7 %, P>0.05) or in IFG plus I-2hPG (25.6% versus 37.8%) (*χ*^2^=0.137, P=0.93). No significant differences were found between the GlucoVita and placebo groups for the distribution of BMI status (normal weight (30.2% versus 26.7%, P>0.05), overweight (46.5% versus 48.9%, P>0.05), obesity (23.3% versus 24.4%, P>0.05), dyslipidemia (39.5% versus 35.6%, P>0.05), hypertension (67.4% versus 71.1%, P>0.05), and self-reported diabetes-related symptoms (46.51% versus 46.67%, P>0.05) (Table 1).

### 3.2. Fasting Glucose

There were no significant differences between the GlucoVita and placebo groups for baseline fasting glucose levels (6.05±0.57 mmol/L versus 6.11±0.51 mmol/L) (P>0.05) or for fasting glucose levels after the 12-week intervention (6.03±0.67mmol/L versus 6.01±0.64 mmol/L) (P>0.05). No significant effects of GlucoVita were found in the stratified subgroups for age (≤65 versus > 65), metabolic symptoms (no or yes), BMI status (normal, overweight, obese), and FG and 2h-PG (normal or impaired) (Table 2) (P>0.05, each). However, 12-week administration of GlucoVita supplements resulted in a significantly reduced fasting glucose level in individuals with normal 2h-PG levels (6.37±0.27 versus 6.08±0.38 mmol/L, P<0.05) but not in individuals with impaired 2h-PG levels (Table 2).

### 3.3. Postprandial Glucose Response

Two-hour postprandial glucose levels (2h-PG) were significantly reduced after 12-week administration of GlucoVita supplements (7.35±2.06 versus 8.15±1.67 mmol/L, P<0.05). No significant differences were found between the GlucoVita and placebo groups for baseline 2h-PG levels (8.15±1.67 versus 7.94±1.54 mmol/L) (P>0.05) and postintervention 2h-PG levels (7.35±2.06 versus 7.41±2.11 mmol/L) (P>0.05) (Table 3).

Stratified subgroup analysis showed that 12-week administration of GlucoVita significantly improved 2h-PG in individuals ≤65 years of age (7.1±2.16 versus 8.02±1.78 mmol/L, P<0.05 for GlucoVita; 7.06±1.95 versus 7.95±1.6, mmol/L, P<0.05, for placebo), in the symptom-free GlucoVita subgroup (7.06±2.01 versus 8.05±1.93 mmol/L, P<0.05), in overweight GlucoVita subgroup (7.2±1.83 versus 8.8±1.51 mmol/L, P<0.05), in individuals with normal fasting glucose ( 7.37±2.14 versus 9.13±1.05 mmol/L, P<0.05 for GlucoVita; and 6.94±1.74 versus 8.65±0.93 mmol/L, P<0.05, for placebo), and in individuals with impaired 2h-PG levels (8.01±1.99 versus 9.2±1.02 mmol/L, P<0.05 for GlucoVita and; 7.7±2.34 versus 8.85±0.99 mmol/L, P<0.05 for placebo).

Of note, overweight individuals in the GlucoVita subgroup had significantly greater baseline 2h-PG levels compared to overweight individuals in the placebo subgroup (8.8±1.51 versus 7.53±1.4 mmol/L, P<0.01), as well as a significantly greater difference in 2h-PG levels after the 12-week GlucoVita administration (1.6±2.12 versus 0.52±1.22 mmol/L, P<0.05, respectively) (Table 3).

### 3.4. HbA1c Levels

No baseline differences in HbA1c levels were found between the GlucoVita and placebo groups (5.85±0.41 versus 5.92±0.42, P>0.05). However, after the intervention, HbA1c levels were lower in the GlucoVita group compared to the placebo group at a trend level (5.81±0.49 versus 6.0±0.51, P=0.08). Similarly, the group difference for the rate of change in HbA1c levels was at a trend level (0.05±0.31 versus -0.08±0.36, P=0.09).

Stratified analysis showed that, for the ≤65-year subgroup, HbA1c levels were slightly but significantly reduced in the GlucoVita subgroup after the 12-week intervention (5.73±0.43 versus 5.85±0.37, P<0.05) but were slightly increased in the placebo group (5.96±0.48 versus 5.89±0.39). HbA1c levels was lower at a trend level in the GlucoVita group compared to the placebo group for the ≤65-year subgroup (5.73±0.43 versus 5.96±0.48, P=0.06) after the 12-week intervention (Table 4).

Similarly, within the metabolic symptom-free subgroups, HbA1c levels were significantly lower in the GlucoVita group compared to the placebo group after the 12-week intervention (5.73±0.45 versus 6.04±0.52, P<0.05) (Table 4). Within the IFG subgroup, HbA1c levels were significantly lower in the GlucoVita group compared to the placebo group after the 12-week intervention (5.85±0.46 versus 6.14±0.5, P<0.05). For the normal 2h-PG subgroup, HbA1c levels were lower in the GlucoVita group compared to the placebo group after the 12-week intervention (5.74±0.41 versus 5.99±0.36, P=0.08) (Table 4).

The rate of reduction of HbA1c levels was greater in the GlucoVita group compared to the placebo group after the 12-week intervention at a trend level (0.05±0.31 versus -0.08±0.36 %, P<0.10). The group differences were significant for ≤65 years of age subgroup (0.12±0.28 versus -0.07±0.41%, P<0.05) (Table 4), the symptom-free subgroup (0.07±0.3 versus -0.15±0.33 %, P<0.05) (Table 4), the overweight subgroups (0.1±0.29 versus -0.09±0.25 %, P<0.05) (Table 4), the impaired FG subgroups (0.08±0.31 versus -0.13±0.38, P<0.05) (Table 6), and the impaired 2h-PG subgroups (0.12±0.34 versus -0.15±0.32, P<0.05) (Table 4).

### 3.5. Correlations between the Variables

Correlation analysis showed no significant association between baseline fasting glucose levels and other variables. However, baseline 2h-PG levels were significantly correlated with body weight, waist circumference, and BMI for all study participants (n=88, r=0.25, P<0.05; r=0.31, P<0.01; r=0.22, P<0.05, respectively) and in the placebo group (r=0.45, P<0.01; r=0.39, P<0.01; r=0.35, P<0.01, respectively) (Tables 5(a)–5(c)) and were improved after the 12-week intervention for all participants (r=0.28, P<0.01; r=0.30, P<0.01; r=0.26, P<0.05, respectively) and for the placebo group (r=0.37, P<0.05; r=0.34, P<0.05; r=0.32, P<0.05). No significant correlations were observed for individuals in the GlucoVita group.

Similarly, baseline HbA1c levels were significantly correlated with baseline fasting glucose levels for all participants (n=88, r=0.27, P<0.01) and in the placebo group (n=45, r=0.36, P<0.05), but only at a trend level in the GlucoVita group (n=43, r=0.19, P>0.05). No significant correlations were found between baseline HbA1c levels and baseline postprandial glucose levels (2h-PG) for all the groups.

However, after the 12-week intervention, HbA1c levels was significantly correlated with FG and 2h-PG in the combined group (n=88, r=0.55, P<0.01; r=0.37, P<0.01), in the placebo group (n=45, r=0.59, P<0.01; r=0.41, P<0.01), and in the GlucoVita group (n=43, r=0.54, P<0.01; r=0.31, P<0.05) (Tables 6(a)–6(c)).

Twelve-week intervention also significantly improved the correlation between fasting glucose levels and postprandial glucose levels for all participants (n=88, from r=-0.103, P> 0.05, to r=0.32, P<0.01), for the GlucoVita group (n=43, from r= -0.191, P>0.05; to r=0.39, P<0.01), but less in the placebo group (n=45, changed from r= 0.01, P>0.05; to r=0.26, P>0.05).

## 4. Discussion

Prediabetes and type 2 diabetes have become a global epidemic, with people having prediabetes being undiagnosed and progressing towards diabetes. Hence there is an urgent need for early diagnosis and effective interventions to prevent and/or reverse the progression of prediabetes in to full blown diabetes. FG, OGTT, and HbA1c are the choice methods to determine abnormal glucose regulation. However, OGTT is too inconvenient of a method for many people and FG and HbA1c are not accurate predictors of prediabetes. Additionally, there are discordances between the results from these tests. Recent studies have shown that new biomarkers for food intake and nutrient status are associated with glucose tolerance status and the development of diabetes [7]. However, validation of any new biomarkers is a long and expensive process.

In this study, we found that postprandial glucose levels are closely associated with the risk factors for diabetes compared to FG and HbA1c levels in elderly individuals with prediabetes. Additionally, a significant reduction in 2h-PG levels and a trend reduction in HbA1c were found after 12-week administration of GlucoVita supplements. These beneficial effects of GlucoVita were primarily observed in individuals ≤ 65 years of age and in metabolic symptom-free individuals. GlucoVita supplements also significantly reduced FG levels in individuals with normal 2h-PG levels and reduced 2h-PG levels in individuals ≤ 65 years of age (but not in individuals >65 year). 2h-PG levels were also reduced in the normal FG subgroup but not in the impaired FG subgroup, suggesting that GlucoVita was more effective in individuals with early stage prediabetes.

Similarly, the beneficial effect of GlucoVita on HbA1c levels was significant only in individuals ≤ 65 years of age and in those without IGT and metabolic symptoms. These results indicate that polyherbal supplementation could be beneficial for the “relatively young” individuals during the early stages of prediabetes. Our results were in agreement with the recent study that indicated daily intake of cinnamon for 12 weeks reduced fasting blood glucose and HbA1c levels among middle-aged type 2 diabetic patients [36]. Although the underlying mechanisms remain to be elucidated, these antidiabetic effects of these herbal supplements may be through increasing glucose uptake and improving insulin sensitivity in peripheral tissues, as well as improving glycogen synthesis in the liver, restoring pancreatic islets dysfunction, slowing down gastric emptying rates, and improving diabetic renal and brain disorders, via multiple signaling pathways, including those of the PPARs, AMPK, PI3K/IRS-1, RBP4-GLUT4, and Nrf2 pathways [37].

In this study, we also found that baseline and postintervention HbA1c levels were significantly correlated with FG levels (r=0.28, P<0.01; r=0.55, P<0.01, respectively) rather than with 2h-PG levels (r=0.14, P>0.05; r=0.37, P<0.01, respectively) or other risk factors in elderly individuals with prediabetes. This supports the notion that HbA1c levels are a good indicator of FG. HbA1c is an index of average glucose over the preceding weeks-to-months and is a more stable indicator of glucose status rather than fasting glucose or postprandial glucose. This is because the erythrocyte (red blood cell) lifespan averages about 120 days. The HbA1c levels at any time point are a result of all circulating erythrocytes, from the oldest (120 days old) to the newly generated. Because HbA1c is a weighted average of blood glucose levels of the preceding 120 days (the lifespan of erythrocytes), newly generated erythrocytes contribute more to determining current glucose levels in the immediate preceding days compared to erythrocytes generated 90-120 days earlier. Thus, HbA1c levels could increase or decrease relatively quickly following a clinically significant change in acute glucose levels.

The known risk factors of prediabetes such as body weight, waist circumference, and BMI are significantly correlated with baseline and postintervention 2h-PG levels rather than with FG or HbA1c. Our results are in-line and suggest that 2h-PG rather than FG or HbA1c should be used as the more reliable indicator to screen elderly individuals with prediabetes. HbA1c levels became highly correlated with FG levels (r=0.53, P<0.01) and 2h-PG levels (r=0.31, P<0.01) after 12-week administration with GlucoVita, supporting a beneficial role of GlucoVita supplements for elderly individuals. 

*Limitations.* There were several limitations of this pilot study and our results should be interpreted with caution. The number of study participants was small and the observational follow-up period was short to comprehensively demonstrate that 2h-PG levels was a better indicator compared to FG and HbA1c levels in predicting the development of diabetes for the different subgroups (age, body weight, symptoms, and BMI) of elderly individuals with prediabetes. Further studies are required to investigate if 2h-PG is more sensitive and reliable compared to FG/HbA1c levels during the prediabetic stage to predict the development of diabetes. In addition, further studies should be performed to determine if herbal supplements could be effective to improve 2h-PG levels at the preclinical stage of diabetes.

## 5. Conclusions

In this study, 2h-PG but not FG and HbA1c levels were significantly correlated with body weight, waist circumference, and BMI (the known risk factors of diabetes in elderly individuals with prediabetes). Twelve-week administration of GlucoVita supplements significantly improved 2h-PG levels only in prediabetics who were symptom-free and were under 65 years of age. As most prediabetics would progress to diabetes in less than 10 years, our study demonstrated a potential benefit of herbal supplements on 2h-PG levels for the management of preclinical stage of diabetes.

## Figures and Tables

**Table 1:** Basic characteristics of the study participants who completed the 12-week study ^a^ (N=88).

	GlucoVita	Placebo	Total	chi-square or t value	*P*
	(N=43)	(N=45)			
Gender					
Male	17(39.5%)	15(33.3%)	32 (36.4%)	0.365	0.545
Female	26(60.5%)	30(66.7%)	56 (63.6%)		
Age, years (mean±s.d.)					
Male	63.2±7.53	67.71±7.27	65.31±7.64		
Female	63.81±8.60	60.69±6.82	62.14±7.79		
Total	63.57±8.11	63.03±7.66	63.29±7.84	0.320	0.750
Age, years (range)					
Male	53.05-78.18	52.38-78.24	52.38-78.24		
Female	52.29-78.52	49.38-78.56	49.38-78.56		
Status					
IFG	16(37.2%)	16(35.6%)	32(36.4%)	1.813	0.404
I2hPG	16(37.2%)	12(26.7%)	28(31.8%)		
IFG&I2hPG	11(25.6%)	17(37.8%)	28(31.8%)		
BMI					
Normal	13(30.2%)	12(26.7%)	25(28.4%)	0.137	0.934
Overweight	20(46.5%)	22(48.9%)	42(47.7%)		
Obese	10(23.3%)	11(24.4%)	21(23.9%)		
BMI (kg/m^2^) (mean±s.d.)					
	25.64±3.42	25.63±3.30	25.64±3.34	0.009	0.993
Weight (kg) (mean±s.d.)					
	64.23±9.89	65.63±11.28	64.95±10.59	-0.618	0.538
Hyperlipidemia					
No	26(60.5%)	29(64.4%)	55(62.5%)	0.149	0.700
Yes	17(39.5%)	16(35.6%)	33(37.5%)		
Hypertension					
No	14(32.6%)	13(28.9%)	27(30.7%)	0.139	0.709
Yes	29(67.4%)	32(71.1%)	61(69.3%)		
Duration of disease, years (mean±s.d.)					
	2.87±3.89	2.34±1.42	2.60±2.90	0.855	0.395
Symptoms					
No	23(53.49%)	24(53.33%)	47(53.4%)	0.0002	0.988
yes	20(46.51%)	21(46.67%)	41(46.6%)		

^a^ Data are numbers of individuals (%) unless otherwise indicated.

**Table 2:** Differences in fasting glucose levels (FG, mmol/l) between the GlucoVita and placebo subgroups stratified by age, BMI, and metabolic symptoms.

Subgroups	Treatment	Preintervention	Postintervention	Change in value
Total				
	GlucoVita (N=43)	6.05±0.57	6.03±0.67	0.02±0.65
	Placebo (N=45)	6.11±0.51	6.01±0.64	0.10±0.46
	t	-0.52	0.15	-0.68
	*P*	0.60	0.88	0.50
AGE				
Age<=65yrs	GlucoVita (N=31)	6.12±0.54	6.01±0.56	0.11±0.49
	Placebo (N=30)	6.11±0.49	6.04±0.63	0.08±0.45
	t	0.03	-0.18	0.25
	*P*	0.98	0.86	0.80
Age>65yrs	GlucoVita (N=12)	5.88±0.63	6.08±0.92	-0.20±0.94
	Placebo (N=15)	6.10±0.56	5.95±0.67	0.15±0.48
	t	-0.99	0.42	-1.27
	*P*	0.33	0.68	0.22
SYMPTONS				
No	GlucoVita (N=23)	6.10±0.55	6.01±0.56	0.09±0.39
	Placebo (N=24)	6.14±0.45	5.98±0.63	0.17±0.48
	t	-0.28	0.19	-0.60
	*P*	0.78	0.85	0.55
Yes	GlucoVita (N=20)	5.99±0.6	6.05±0.79	-0.06±0.87
	Placebo (N=21)	6.07±0.57	6.04±0.66	0.03±0.43
	t	-0.44	0.03	-0.41
	*P*	0.66	0.97	0.68
BMI				
Normal	GlucoVita (N=13)	6.17±0.45	6.10±0.37	0.07±0.30
	Placebo (N=12)	6.06±0.55	6.23±0.75	-0.16±0.56
	t	0.53	-0.53	1.30
	*P*	0.60	0.60	0.21
Overweight	GlucoVita (N=20)	5.99±0.58	5.95±0.46	0.04±0.44
	Placebo (N=22)	6.01±0.53	5.75±0.5	0.26±0.36
	t	-0.13	1.32	-1.76
	*P*	0.90	0.20	0.09
Obese	GlucoVita (N=10)	6.03±0.71	6.1±1.19	-0.08±1.21
	Placebo (N=11)	6.37±0.33	6.28±0.6	0.09±0.39
	t	-1.43	-0.45	-0.42
	*P*	0.17	0.66	0.68
FG				
Normal	GlucoVita (N=16)	5.46±0.41	5.73±0.77	-0.27±0.79
	Placebo (N=12)	5.46±0.43	5.33±0.21	0.13±0.47
	t	-0.03	1.71	-1.55
	*P*	0.98	0.10	0.13
Impaired	GlucoVita (N=27)	6.40±0.29	6.21±0.53	0.19±0.5
	Placebo (N=33)	6.35±0.27	6.25±0.56	0.09±0.46
	t	0.77	-0.31	0.81
	*P*	0.44	0.76	0.42
2h-PG				
Normal	GlucoVita (N=16)	6.37±0.27	6.08±0.38*∗*	0.29±0.48
	Placebo (N=16)	6.25±0.20	6.21±0.53	0.05±0.45
	t	1.35	-0.80	1.50
	*P*	0.19	0.43	0.14
Impaired	GlucoVita (N=27)	5.86±0.62	6.00±0.79	-0.14±0.7
	Placebo (N=29)	6.03±0.6	5.90±0.67	0.14±0.46
	t	-1.04	0.53	-1.74
	*P*	0.30	0.60	0.09

*∗*, P<0.05 within-group comparison preintervention versus postintervention.

**Table 3:** Differences in 2h-postprandial glucose levels (2h-PG, mmol/l) between the GlucoVita and placebo subgroups stratified by age, BMI, and metabolic symptoms.

Subgroup	Treatment	Preintervention	Postintervention	Change in Value
Total				
	GlucoVita (N=43)	8.15±1.67	7.35±2.06*∗*	0.76±2.02
	Placebo (N=45)	7.94±1.54	7.41±2.11	0.60±1.92
	t	0.61	-0.13	0.39
	*P*	0.55	0.90	0.69
AGE				
Age<=65yrs	GlucoVita (N=31)	8.02±1.78	7.10±2.16*∗*	0.92±2.00
	Placebo (N=30)	7.95±1.6	7.06±1.95*∗*	0.99±1.93
	t	0.17	0.08	-0.13
	*P*	0.86	0.94	0.90
Age>65yrs	GlucoVita (N=12)	8.48±1.37	8.00±1.7	0.36±2.11
	Placebo (N=15)	7.93±1.48	8.11±2.31	-0.18±1.71
	t	0.99	-0.13	0.73
	*P*	0.33	0.89	0.47
SYMPTOMS				
No symptoms	GlucoVita (N=23)	8.05±1.93	7.06±2.01*∗*	0.99±2.16
	Placebo (N=24)	7.41±1.41	6.98±1.76	0.42±1.41
	t	1.30	0.14	1.06
	*P*	0.20	0.89	0.29
Symptoms present	GlucoVita (N=20)	8.27±1.36	7.69±2.12	0.51±1.87
	Placebo (N=21)	8.55±1.49	7.90±2.41	0.80±2.4
	t	-0.64	-0.30	-0.43
	*P*	0.53	0.77	0.67
BMI				
Normal	GlucoVita (N=13)	7.33±1.68	7.05±2.23	0.18±1.91
	Placebo (N=12)	7.6±1.11	6.68±1.49	0.93±2.24
	t	-0.47	0.48	-0.91
	*P*	0.64	0.63	0.37
Overweight	GlucoVita (N=20)	8.80±1.51^++^	7.20±1.83*∗*	1.60±2.12^+^
	Placebo (N=22)	7.53±1.4	7.15±1.82	0.52±1.22
	t	2.83	0.10	2.05
	*P*	0.01	0.92	0.05
Obese	GlucoVita (N=10)	7.90±1.57	8.05±2.32	-0.15±1.31
	Placebo (N=11)	9.14±1.7	8.74±2.74	0.40±2.72
	t	-1.72	-0.62	-0.58
	*P*	0.10	0.55	0.57
FG				
Normal	GlucoVita (N=16)	9.13±1.05	7.37±2.14*∗*	1.76±2.67
	Placebo (N=12)	8.65±0.93	6.94±1.74*∗*	1.71±2.02
	t	1.25	0.57	0.06
	*P*	0.22	0.58	0.96
Impaired	GlucoVita (N=27)	7.57±1.71	7.34±2.06	0.18±1.24
	Placebo (N=33)	7.68±1.65	7.58±2.23	0.20±1.75
	t	-0.26	-0.43	-0.05
	*P*	0.79	0.67	0.96
2h-PG				
Normal	GlucoVita (N=16)	6.37±0.81	6.24±1.7	0.05±1.40
	Placebo (N=16)	6.30±0.85	6.88±1.54	-0.39±0.9
	t	0.24	-1.12	1.05
	*P*	0.81	0.27	0.30
Impaired	GlucoVita (N=27)	9.20±1.02	8.01±1.99*∗*	1.19±2.23
	Placebo (N=29)	8.85±0.99	7.70±2.34*∗*	1.15±2.12
	t	1.33	0.53	0.08
	*P*	0.19	0.60	0.94

*∗*, P<0.05 within-group comparison: preintervention versus postintervention.

^+^, P<0.05 between-group comparison.

^++^, P<0.01 between-group comparison.

**Table 4:** Differences in HbA1c levels (%) between the GlucoVita and placebo subgroups stratified by age, BMI, and metabolic symptoms.

Subgroups	Treatment	Preintervention	Postintervention	Change in Value
Total				
	GlucoVita (N=43)	5.85±0.41	5.81±0.49	0.05±0.31
	Placebo (N=45)	5.92±0.42	6.00±0.51	-0.08±0.36
	t	-0.76	-1.80	1.73
	*P*	0.45	0.08	0.09
AGE				
Age<=65yrs	GlucoVita (N=31)	5.85±0.37	5.73±0.43*∗*	0.12±0.28^+^
	Placebo (N=30)	5.89±0.39	5.96±0.48	-0.07±0.41
	t	-0.36	-1.92	2.10
	*P*	0.72	0.06	0.04
Age>65yrs	GlucoVita (N=12)	5.86±0.51	6.00±0.58	-0.14±0.31
	Placebo (N=15)	5.99±0.47	6.08±0.57	-0.09±0.23
	t	-0.68	-0.36	-0.46
	*P*	0.50	0.72	0.65
SYMPTOMS				
No	GlucoVita (N=23)	5.80±0.37	5.73±0.45^+^	0.07±0.30^+^
	Placebo (N=24)	5.89±0.43*∗*	6.04±0.52	-0.15±0.33
	t	-0.78	-2.14	2.29
	*P*	0.44	0.04	0.03
YES	GlucoVita (N=20)	5.92±0.45	5.89±0.53	0.02±0.33
	Placebo (N=21)	5.95±0.41	5.95±0.51	0.00±0.38
	t	-0.28	-0.39	0.22
	*P*	0.78	0.70	0.82
BMI				
Normal	GlucoVita (N=13)	5.86±0.39	5.82±0.43	0.04±0.32
	Placebo (N=12)	5.98±0.38	6.03±0.62	-0.06±0.30
	t	-0.74	-0.99	0.78
	*P*	0.47	0.33	0.44
Overweight	GlucoVita (N=20)	5.75±0.38	5.65±0.37	0.10±0.29
	Placebo (N=22)	5.75±0.36	5.84±0.37	-0.09±0.25
	t	0.00	-1.62	2.22
	*P*	1.00	0.11	0.03
Obese	GlucoVita (N=10)	6.06±0.44	6.10±0.64	-0.04±0.35
	Placebo (N=11)	6.21±0.42	6.28±0.53	-0.07±0.58
	t	-0.79	-0.71	0.15
	*P*	0.44	0.49	0.88
FG				
Normal	GlucoVita (N=16)	5.73±0.47	5.73±0.53	-0.01±0.32
	Placebo (N=12)	5.68±0.26	5.60±0.27	0.07±0.24
	t	0.36	0.78	-0.75
	*P*	0.72	0.44	0.46
Impaired	GlucoVita (N=27)	5.93±0.35	5.85±0.46^+^	0.08±0.31^+^
	Placebo (N=33)	6.01±0.43	6.14±0.5	-0.13±0.38
	t	-0.77	-2.32	2.31
	*P*	0.44	0.02	0.02
2h-PG				
Normal	GlucoVita (N=16)	5.86±0.36	5.74±0.41	0.12±0.34^+^
	Placebo (N=16)	5.84±0.29	5.99±0.36	-0.15±0.32
	t	0.16	-1.83	2.30
	*P*	0.87	0.08	0.03
Impaired	GlucoVita (N=27)	5.85±0.44	5.85±0.53	0.00±0.29
	Placebo (N=29)	5.97±0.47	6.00±0.58	-0.04±0.38
	t	-0.93	-1.04	0.46
	*P*	0.36	0.30	0.65

*∗*, P<0.05 within-group comparison: preintervention versus postintervention.

^+^, P<0.05 between-group comparison.

**Table:** (a) Baseline correlations of the pooled study participants (N=88)

	Age	Illness duration	Height	Weight	Waist	Hip	BMI	W/H ratio	FG	2h-PG	HbA1c
Age	1										
Duration of disease	-0.087	1									
Height	-0.108	.242*∗*	1								
Weight	-0.049	0.065	.604*∗∗*	1							
Waist	0.008	-0.114	.277*∗∗*	.766*∗∗*	1						
Hip	0.189	0.028	.244*∗*	.725*∗∗*	.778*∗∗*	1					
BMI	0.033	-0.115	-0.038	.769*∗∗*	.709*∗∗*	.688*∗∗*	1				
W/H ratio	-0.195	-.213*∗*	0.159	.409*∗∗*	.721*∗∗*	0.127	.368*∗∗*	1			
FG	-0.19	0.17	0.139	0.143	0.063	0.057	0.057	0.018	1		
2h-PG	0.101	0.008	0.097	.247*∗*	.311*∗∗*	0.205	.217*∗*	.276*∗∗*	-0.103	1	
HbA1c	0.046	-0.101	-0.09	0.08	0.104	0.106	0.152	0.04	.276*∗∗*	0.136	1

*∗*, Pearson correlation was significant at the 0.05 level (2-tailed).

*∗∗*, Pearson correlation was significant at the 0.01 level (2-tailed).

**Table:** (b) Baseline correlations within the GlucoVita group (N=43)

	Age	Illness duration	Height	Weight	Waist	Hip	BMI	W/H ratio	FG	2h-PG	HbA1c
Age	1										
Duration of disease	-0.143	1									
Height	-.305*∗*	.354*∗*	1								
Weight	-0.213	0.173	.556*∗∗*	1							
Waist	-0.008	-0.061	0.218	.670*∗∗*	1						
Hip	0.232	0.058	0.105	.534*∗∗*	.782*∗∗*	1					
BMI	0.006	-0.093	-0.184	.708*∗∗*	.559*∗∗*	.497*∗∗*	1				
W/H ratio	-0.27	-0.16	0.211	.454*∗∗*	.704*∗∗*	0.111	.334*∗*	1			
FG	-0.284	0.155	0.286	0.137	-0.15	-0.159	-0.076	-0.066	1		
2h-PG	0.157	0.031	-0.095	0.033	0.237	0.057	0.095	.331*∗*	-0.191	1	
HbA1c	-0.094	-0.105	-0.228	-0.049	-0.035	-0.058	0.116	0.022	0.193	0.093	1

*∗*, Pearson correlation was significant at the 0.05 level (2-tailed).

*∗∗*, Pearson correlation was significant at the 0.01 level (2-tailed).

**Table:** (c) Baseline correlations within the placebo group (N=45)

	Age	Illness duration	Height	Weight	Waist	Hip	BMI	W/H ratio	FG	2h-PG	HbA1c
Age	1										
Duration of disease	0.023	1									
Height	0.109	0.056	1								
Weight	0.099	-0.127	.647*∗∗*	1							
Waist	0.024	-0.267	.323*∗*	.829*∗∗*	1						
Hip1	0.156	-0.02	.366*∗*	.859*∗∗*	.777*∗∗*	1					
BMI	0.061	-0.218	0.116	.830*∗∗*	.840*∗∗*	.851*∗∗*	1				
W/H ratio	-0.14	-.415*∗∗*	0.109	.374*∗*	.728*∗∗*	0.137	.404*∗∗*	1			
FG	-0.08	0.293	-0.039	0.144	0.238	0.249	0.205	0.081	1		
2h-PG	0.035	-0.08	.323*∗*	.455*∗∗*	.388*∗∗*	.336*∗*	.348*∗*	0.252	0.007	1	
HbA1c	0.19	-0.103	0.03	0.176	0.199	0.232	0.188	0.042	.359*∗*	0.192	1

*∗*, Pearson correlation was significant at the 0.05 level (2-tailed).

*∗∗*, Pearson correlation was significant at the 0.01 level (2-tailed).

**Table:** (a) Correlations of the pooled groups after the 12-week intervention (N=88)

	Age	Illness duration	Height	Weight	Waist	Hip	BMI	W/H ratio	FG	2h-PG	HbA1c
Age	1										
Duration of Illness	-0.087	1									
Height	-0.125	.238*∗*	1								
Weight	-0.068	0.073	.664*∗∗*	1							
Waist	0.046	-0.111	.314*∗∗*	.839*∗∗*	1						
Hip	0.142	-0.001	.266*∗*	.793*∗∗*	.843*∗∗*	1					
BMI	0.006	-0.099	0.111	.808*∗∗*	.868*∗∗*	.858*∗∗*	1				
W/H ratio	-0.092	-0.199	.225*∗*	.526*∗∗*	.754*∗∗*	.284*∗∗*	.510*∗∗*	1			
Fasting glucose	-0.047	0.179	0.064	0.043	0.033	0.057	0.004	-0.029	1		
2h-PG	.280*∗∗*	0.097	0.116	.278*∗∗*	.303*∗∗*	.344*∗∗*	.257*∗*	0.115	.326*∗∗*	1	.370*∗∗*
HbA1c	0.197	0.045	-0.132	-0.011	0.062	0.094	0.073	-0.018	.553*∗∗*	.370*∗∗*	1

*∗*, Pearson correlation was significant at the 0.05 level (2-tailed).

*∗∗*, Pearson correlation was significant at the 0.01 level (2-tailed).

**Table:** (b) Correlations within the GlucoVita group after the 12-week intervention (N=43)

	Age	Illness duration	Height	Weight	Waist	Hip	BMI	W/H ratio	FG	2h-PG	HbA1c
Age	1										
Duration of Illness	-0.143	1									
Height	-.325*∗*	.348*∗*	1								
Weight	-0.208	0.189	.689*∗∗*	1							
Waist	0.029	-0.058	0.26	.785*∗∗*	1						
Hip	0.213	0.045	0.123	.678*∗∗*	.832*∗∗*	1					
BMI	0.004	-0.069	0.057	.747*∗∗*	.850*∗∗*	.836*∗∗*	1				
W/H ratio	-0.195	-0.152	0.289	.553*∗∗*	.752*∗∗*	0.261	.495*∗∗*	1			
Fasting glucose	-0.012	0.154	0.129	0.097	0.017	0.034	0.017	-0.001	1		
2h-PG	0.216	0.068	-0.027	0.157	0.261	0.215	0.181	0.197	.395*∗∗*	1	
HbA1c	0.181	0.016	-0.225	-0.056	0.041	0.069	0.104	-0.002	.538*∗∗*	.309*∗*	1

*∗*, Pearson correlation was significant at the 0.05 level (2-tailed).

*∗∗*, Pearson correlation was significant at the 0.01 level (2-tailed).

**Table:** (c) Correlations within the placebo group after the 12-week intervention (N=45)

	Age	Illness duration	Height	Weight	Waist	Hip	BMI	W/H ratio	FG	2h-PG	HbA1c
Age	1										
Duration of Illness	0.023	1									
Height	0.092	0.07									
Weight	0.059	-0.112	.642*∗∗*	1							
Waist	0.066	-0.257	.353*∗*	.874*∗∗*	1						
Hip	0.088	-0.085	.380*∗∗*	.872*∗∗*	.850*∗∗*	1					
BMI	0.012	-0.197	0.147	.849*∗∗*	.879*∗∗*	.872*∗∗*	1				
W/H ratio	0.007	-.380*∗∗*	0.157	.500*∗∗*	.754*∗∗*	.297*∗*	.518*∗∗*	1			
Fasting glucose	-0.085	.305*∗*	0.001	0.001	0.049	0.078	-0.004	-0.052	1		
2h-PG	.345*∗*	0.217	0.254	.375*∗*	.337*∗*	.445*∗∗*	.316*∗*	0.044	0.26	1	
HbA1c	0.233	0.211	-0.089	-0.011	0.054	0.1	0.032	-0.059	.595*∗∗*	.431*∗∗*	1

*∗*, Pearson correlation was significant at the 0.05 level (2-tailed).

*∗∗*, Pearson correlation was significant at the 0.01 level (2-tailed).

## Data Availability

No additional data are available.

## Abbreviations

FG: Fasting glucose level
GlucoVita: A polyherbal formulation (for prediabetics)
HbA1c: Glycosylated hemoglobin
IFG: Impaired fasting glucose level
IGT: Impaired glucose tolerance
2h-PG: 2-hour postprandial glucose level.

## References

[1] Anjana R. M., Shanthi Rani C. S., Deepa M. (2015). Incidence of diabetes and prediabetes and predictors of progression among asian indians: 10-year follow-up of the chennai urban rural epidemiology study (CURES). *Diabetes Care*.

[2] Centers for Disease Control and Prevention (CDC) (2013). Awareness of prediabetes--United States, 2005-2010. *MMWR: Morbidity and Mortality Weekly Report*.

[3] Grundy S. M. (2012). Pre-diabetes, metabolic syndrome, and cardiovascular risk. *Journal of the American College of Cardiology*.

[4] Ho-Pham L. T., Nguyen U. D. T., Tran T. X., Nguyen T. V. (2017). Discordance in the diagnosis of diabetes: comparison between HbA1c and fasting plasma glucose. *PLoS One*.

[5] Lo C. C., Lara J., Cheng T. C. (2017). Skin deep: enhanced variable may help explain racial disparities in type 2 diabetes and prediabetes. *Diabetes Therapy*.

[6] Lopez Lopez R., Fuentes Garcia R., Gonzalez-Villalpando M. E., Gonzalez-Villalpando C. (2017). Diabetic by HbA1c, normal by OGTT: a frequent finding in the mexico city diabetes study. *Journal of the Endocrine Society*.

[7] Savolainen O., Lind M. V., Bergström G., Fagerberg B., Sandberg A.-S., Ross A. (2017). Biomarkers of food intake and nutrient status are associated with glucose tolerance status and development of type 2 diabetes in older Swedish women. *American Journal of Clinical Nutrition*.

[8] Agarwal A., Hegde A., Yadav C., Ahmad A., Manjrekar P. A., Srikantiah R. M. (2016). Assessment of oxidative stress and inflammation in prediabetes—A hospital based cross-sectional study. *Diabetes & Metabolic Syndrome: Clinical Research & Reviews*.

[9] Lotfy M., Adeghate J., Kalasz H., Singh J., Adeghate E. (2017). Chronic complications of diabetes mellitus: a mini review. *Current Diabetes Reviews*.

[10] Sotoudeh G., Abshirini M., Bagheri F., Siassi F., Koohdani F., Aslany Z. (2018). Higher dietary total antioxidant capacity is inversely related to prediabetes: A case-control study. *Nutrition Journal *.

[11] Bhatti J. S., Bhatti G. K., Reddy P. H. (2017). Mitochondrial dysfunction and oxidative stress in metabolic disorders — A step towards mitochondria based therapeutic strategies. *Biochimica et Biophysica Acta (BBA) - Molecular Basis of Disease*.

[12] Haxhi J., Leto G., di Palumbo A. S. (2016). Exercise at lunchtime: effect on glycemic control and oxidative stress in middle-aged men with type 2 diabetes. *European Journal of Applied Physiology*.

[13] Halmos T., Suba I. (2016). Alzheimer's disease and diabetes - the common pathogenesis. *Neuropsychopharmacologia Hungarica*.

[14] Jaruratanasirikul S., Thammaratchuchai S., Puwanant M., Mo-suwan L., Sriplung H. (2016). Progression from impaired glucose tolerance to type 2 diabetes in obese children and adolescents: a 3–6-year cohort study in southern Thailand. *Journal of Pediatric Endocrinology and Metabolism*.

[15] Arslan M. S., Tutal E., Sahin M. (2017). Effect of lifestyle interventions with or without metformin therapy on serum levels of osteoprotegerin and receptor activator of nuclear factor kappa B ligand in patients with prediabetes. *Endocrine Journal*.

[16] Eaglehouse Y. L., Venditti E. M., Kramer M. K. (2017). Factors related to lifestyle goal achievement in a diabetes prevention program dissemination study. *Translational Behavioral Medicine*.

[17] Wennehorst K., Mildenstein K., Saliger B. (2016). A comprehensive lifestyle intervention to prevent type 2 diabetes and cardiovascular diseases: the German CHIP trial. *Prevention Science*.

[18] Burke S. J., Karlstad M. D., Conley C. P., Reel D., Whelan J., Jason Collier J. (2015). Dietary polyherbal supplementation decreases CD3+ cell infiltration into pancreatic islets and prevents hyperglycemia in nonobese diabetic mice. *Nutrition Research*.

[19] Hirotani Y., Okumura K., Yoko U., Myotoku M. (2013). Effects of Gosha-jinki-gan (Chinese herbal medicine: Niu-Che-Sen-Qi-Wan) on hyperinsulinemia and hypertriglyceridemia in prediabetic Zucker fatty rats. *Drug Discoveries & Therapeutics*.

[20] Zhu X., Zhou Y., Xu Q., Wu J. (2017). Traditional Chinese medicine Jianpi Bushen therapy suppresses the onset of pre-metastatic niche in a murine model of spontaneous lung metastasis. *Biomedicine & Pharmacotherapy*.

[21] Teixeira C. C., Fuchs F. D. (2006). The efficacy of herbal medicines in clinical models: The case of jambolan. *Journal of Ethnopharmacology*.

[22] Chaiyasut C., Sivamaruthi B. S., Pengkumsri N. (2016). Germinated Thai black rice extract protects experimental diabetic rats from oxidative stress and other diabetes-related consequences. *Pharmaceuticals*.

[23] Bulboaca A., Bolboacă S. D., Sucis S. (2016). Protective effect of curcumin in fructose-induced metabolic syndrome and in streptozotocin-induced diabetes in rats. *The Iranian Journal of Basic Medical Sciences*.

[24] Zhu D., Wang H., Zhang J. (2015). Irisin improves endothelial function in type 2 diabetes through reducing oxidative/nitrative stresses. *Journal of Molecular and Cellular Cardiology*.

[25] Lo H. Y., Li C. C., Chen F. Y., Chen J. C., Hsiang C. Y., Ho T. Y. (2017). Gastro-resistant insulin receptor-binding peptide from momordica charantia improved the glucose tolerance in streptozotocin-induced diabetic mice via insulin receptor signaling pathway. *Journal of Agricultural and Food Chemistry*.

[26] Ma C., Yu H., Xiao Y., Wang H. (2017). *Momordica charantia* extracts ameliorate insulin resistance by regulating the expression of SOCS-3 and JNK in type 2 diabetes mellitus rats. *Pharmaceutical Biology*.

[27] Mahmoud M. F., El Ashry F. E. Z. Z., El Maraghy N. N., Fahmy A. (2017). Studies on the antidiabetic activities of Momordica charantia fruit juice in streptozotocin-induced diabetic rats. *Pharmaceutical Biology*.

[28] Mahwish, Saeed F., Arshad M. S., Nisa M. U., Nadeem M. T., Arshad M. U. (2017). Hypoglycemic and hypolipidemic effects of different parts and formulations of bitter gourd (Momordica Charantia). *Lipids in Health and Disease*.

[29] Aljohi A., Matou-Nasri S., Ahmed N. (2016). Antiglycation and antioxidant properties of Momordica charantia. *PLoS ONE*.

[30] Yang S. J., Choi J. M., Park S. E. (2015). Preventive effects of bitter melon (Momordica charantia) against insulin resistance and diabetes are associated with the inhibition of NF-*κ*B and JNK pathways in high-fat-fed OLETF rats. *The Journal of Nutritional Biochemistry*.

[31] Xu X., Shan B., Liao C., Xie J., Wen P., Shi J. (2015). Anti-diabetic properties of Momordica charantia L. polysaccharide in alloxan-induced diabetic mice. *International Journal of Biological Macromolecules*.

[32] Wang H.-Y., Kan W.-C., Cheng T.-J., Yu S.-H., Chang L.-H., Chuu J.-J. (2014). Differential anti-diabetic effects and mechanism of action of charantin-rich extract of Taiwanese Momordica charantia between type 1 and type 2 diabetic mice. *Food and Chemical Toxicology*.

[33] Habicht S. D., Ludwig C., Yang R. Y., Krawinkel M. B. (2014). Momordica charantia and type 2 diabetes: From in vitro to human studies. *Current Diabetes Reviews*.

[34] Yin R. V., Lee N. C., Hirpara H., Phung O. J. (2014). The effect of bitter melon (Mormordica charantia) in patients with diabetes mellitus: a systematic review and meta-analysis. *Nutrition & Diabetes*.

[35] Ooi C. P., Yassin Z., Hamid T.-A. (2010). Momordica charantia for type 2 diabetes mellitus. *Cochrane Database of Systematic Reviews (Online)*.

[36] Sahib A. S. (2016). Antidiabetic and antioxidant effect of cinnamon in poorly controlled type-2 diabetic iraqi patients: a randomized, placebo-controlled clinical trial. *Journal of Intercultural Ethnopharmacology*.

[37] Zhu R., Liu H., Liu C. (2017). Cinnamaldehyde in diabetes: a review of pharmacology, pharmacokinetics and safety. *Pharmacological Research*.

[38] Shen T., Xing G., Zhu J. (2017). Effects of 12-week supplementation of marine Omega-3 PUFA-based formulation Omega3Q10 in older adults with prehypertension and/or elevated blood cholesterol. *Lipids in Health and Disease*.

